# Metabolic Responses of *Pleurotus djamor* to Lignocellulosic Substrate Gradients

**DOI:** 10.3390/molecules31193533

**Published:** 2026-10-03

**Authors:** Humberto Raúl Pacheco-Sánchez, Noemí Alitzel Aguado-Rivera, Carlos Eligio Hernández-Navarrete, Amanda Kim Rico-Chávez, Humberto Aguirre-Becerra, Juan Fernando García-Trejo, Ana Angélica Feregrino-Pérez

**Affiliations:** 1 Facultad de Ingeniería, Universidad Autónoma de Querétaro, Amazcala, El Marqués 76265, Querétaro, Mexico; humberto.pacheco@uaq.mx (H.R.P.-S.); noemi.alitzel.ar@gmail.com (N.A.A.-R.); hernandez.navarrete.carlos@gmail.com (C.E.H.-N.); humberto.aguirre@uaq.mx (H.A.-B.); fernando.garcia@uaq.mx (J.F.G.-T.); 2 Facultad de Química, Universidad Autónoma de Querétaro, Cerro de las Campanas, Santiago de Querétaro 76017, Querétaro, Mexico; amanda.rico@uaq.edu.mx

**Keywords:** circular agriculture, residual substrate, fungal physiology, chemometrics, metabolite profiling

## Abstract

*Pleurotus djamor* is cultivated on diverse lignocellulosic residues, but the relationship between progressive substrate formulation, mushroom productivity, and metabolomic responses remains insufficiently characterized. This study evaluated the effects of progressive replacement of barley straw with coconut fiber or coffee husk on mushroom productivity and GC–MS-based metabolomic profiles. Productivity differed markedly among substrate formulations, with intermediate inclusion levels producing the highest yield and biological efficiency. In contrast, PERMANOVA did not detect significant global differences in metabolomic composition across either substrate gradient, and no individual metabolite remained significant after multiple-testing correction. PCA and heatmap analyses revealed exploratory variation in amino acids, carbohydrates, lipids, and related metabolite groups. Pathway analysis identified statistically supported enrichment of alanine, aspartate and glutamate metabolism and cyanoamino acid metabolism in the coffee husk gradient, whereas the remaining pathway associations were exploratory after FDR correction. Overall, the results indicate that substrate composition strongly influenced productivity, while the global metabolomic organization of *P. djamor* remained comparatively stable, with localized metabolite and pathway patterns that require further validation.

## 1. Introduction

Edible mushrooms are widely recognized for their nutritional value and health-promoting properties [1]. They are rich in nutrients such as proteins, carbohydrates, lipids, fiber vitamins and minerals [2]. In addition, edible mushrooms contain bioactive compounds from different chemical classes, including β-glucans, lectins, triterpenoids, nucleosides and phenolic compounds, which are associated with immunomodulatory, anticancer, antioxidant and anti-inflammatory properties [3]. These characteristics have increased their relevance as functional foods and nutraceuticals. Species of the genus *Pleurotus* are notable for their high nutritional value and their capacity to produce bioactive compounds with therapeutic properties [4].

Mushrooms synthesize a wide diversity of metabolites involved in cell differentiation, development, growth, morphology and asexual reproduction [5]. Among these metabolites, amino acids contribute to energy metabolism, cellular homeostasis, nutritional value, and flavor development [6]. Consequently, metabolite composition reflects the physiological state of mushrooms and their cellular responses to environmental and nutritional conditions [6,7]. In particular, fungal metabolism exhibits considerable plasticity, allowing organisms to dynamically adjust metabolic pathways in response to variations in nutrient availability, environmental and physicochemical factors [6,8,9]. Consequently, metabolomic approaches offer valuable opportunities to understand how cultivation conditions shape fungal physiology and biochemical composition.

Mushroom cultivation depends mainly on lignocellulosic substrates, which act as both structural support and nutrient sources [10]. The substrate composition, including cellulose, hemicellulose, and lignin, strongly influences fungal growth, yield and metabolic activity [10,11]. Agro-industrial residues such as coconut fiber, coffee waste, and cereal by-products are widely used due to their availability and sustainability [12,13,14]. Variations in substrate composition can significantly affect fungal physiology and biochemical responses, influencing the quality of the final product [11,15].

Substrate formulation represents a key factor in shaping fungal metabolic profiles and overall product quality [16]. Advances in gas chromatography–mass spectrometry (GC–MS), combined with metabolite profiling approaches, have enabled the comprehensive characterization of these compounds and their relationship with cultivation conditions [15,17,18]. Despite these advances, most studies have focused on comparing substrate types rather than evaluating dose-dependent or gradient-based formulations [15,19].

Recent studies have demonstrated that lignocellulosic substrate composition plays a decisive role in modulating both productivity and metabolic profiles in Pleurotus species. For example, it has been reported that substrate selection influences yield through factors such as the carbon-to-nitrogen ratio, which affects nutrient availability and mycelial growth [20]. Other studies have shown that variations in cultivation substrates can alter the composition of bioactive metabolites, suggesting that substrate-derived nutrients influence fungal metabolic processes [21].

Agro-industrial substrates significantly modulate lignocellulolytic enzyme production, suggesting that substrate-derived components regulate pathways linked to both primary metabolism and substrate degradation [22].

Previous studies have demonstrated that substrate composition can influence mushroom productivity, elemental composition, and the accumulation of bioactive compounds [15,19,20,21,22,23]. Substrate-dependent studies in edible and oyster mushrooms have examined differences among substrate types and supplementation levels in relation to growth, yield, mineral composition, bioactive compounds, and metabolite profiles [15,19,20,21,22,23]. These studies demonstrate that substrate composition is an important determinant of fungal performance and biochemical characteristics. However, most have evaluated individual substrate formulations or supplementation conditions, whereas systematic comparisons of progressive replacement gradients using a common baseline substrate remain less extensively characterized at the metabolomic level [24,25,26].

The specific contribution of the present study is the integrated evaluation of two agro-industrial residue replacement gradients—coconut fiber and coffee husk—using barley straw as a common substrate baseline in *Pleurotus djamor*. The experimental design combined five inclusion levels for each residue with productivity measurements and GC–MS-based metabolomic profiling within the same cultivation framework. Metabolomic responses were examined using multivariate analyses, pathway mapping, and metabolite-level statistical testing, allowing metabolic patterns to be evaluated together with production responses.

This approach provides a comparative framework for determining whether progressive changes in substrate composition are accompanied by statistically supported changes in the metabolome or primarily by exploratory variation while productivity changes. The present study focused on the response of *P. djamor* to progressive changes in substrate composition rather than on directly measuring substrate degradation or modification. Therefore, the aim of this study was to evaluate the metabolomic responses and associated productivity changes in *P. djamor* cultivated on lignocellulosic substrate gradients using GC–MS-based metabolite profiling and multivariate analysis.

## 2. Results and Discussion

### 2.1. Production of Biomass

Yield and biological efficiency varied according to substrate composition, although distinct response patterns were observed between coconut fiber- and coffee husk-based gradients (Figure 1). In coconut fiber-based substrates, yield showed significant differences among treatments (ANOVA, *p* < 0.05); the highest productivity was observed at low-to-moderate inclusion levels (20–40%), where yield values ranged from approximately 16–22% (Figure 1a). In contrast, increasing coconut fiber proportions (60–100%) resulted in a progressive reduction in productivity, with the lowest values observed at 100% inclusion (5.6–11.5%). Biological efficiency followed a similar trend, with the highest values recorded at 20–40% inclusion levels (73.6–101.2%); treatments containing high proportions of coconut fiber exhibited substantially lower biological efficiency values (17.0–65.8%) (Figure 1c).

In coffee husk-based substrates, yield also differed significantly among treatments (Kruskal–Wallis, *p* < 0.05), showing a progressive increase at intermediate-to-high inclusion levels (Figure 1b). The highest productivity was observed at 60–80% coffee husk inclusion, where yield values ranged from approximately 23–51% whereas lower inclusion levels (20–40%) generally showed reduced productivity (10–20%). Biological efficiency exhibited a comparable response pattern, with the highest values likewise recorded at intermediate-to-high inclusion levels, reaching up to 171.9%; lower substrate proportions presented reduced biological efficiency values ranging from 44.2% to 80.8% (Figure 1d).

These contrasting responses indicate that the physicochemical properties of each lignocellulosic residue differentially affected fungal growth. It has been reported that certain substrates can biofortify mushroom production depending on the availability of sugars and essential trace elements such as iron, zinc, and selenium. Key characteristics known to benefit from substrate adjustments include the concentration of active compounds and overall biomass yield [27].

Published studies have reported that coffee husk can contain approximately 40% cellulose, 13% hemicellulose, and 23% Klason lignin, together with several mineral elements [28]. Coffee husk has also been reported to contain phenolic compounds such as gallic acid, chlorogenic acid, and caffeic acid [29]; caffeic acid is associated with fungal growth promotion [30] and chlorogenic acid is reported to exhibit antifungal activity [31]. These reported characteristics provide a useful biochemical context for interpreting the productivity responses observed in the present study. However, because the chemical composition of the substrate batches used here was not directly measured, these literature values should not be considered quantitative measurements of the experimental substrates. Intermediate proportions of coffee husk combined with barley straw were associated with the highest productivity, which may reflect a more favorable combination of substrate characteristics; however, the specific compositional factors responsible were not directly determined in this study.

Similarly, published analyses of coconut fiber have reported approximately 46% lignin, 43% cellulose, and less than 0.5% hemicellulose [32]. This reported composition differs substantially from that described for coffee husk and provides a possible context for the contrasting productivity responses observed between the two substrate systems. However, the present study did not directly quantify lignin, cellulose, hemicellulose, or the carbon-to-nitrogen ratio of the experimental substrates. Therefore, the contribution of these compositional factors to the observed productivity patterns remains a hypothesis rather than a directly tested relationship.

Although fungal degradation of lignin releases bioactive phenolic monomers—such as vanillin, *p*-coumaric acid, ferulic acid, and gallic acid—that can stimulate growth, high substrate inclusion shifts this dynamic [33]. Specifically, the enhanced productivity at 20% and 40% coconut fiber inclusion may be associated with the low-dose effects of bioactive compounds released during initial lignin breakdown. Conversely, the reduction in productivity above 60% inclusion may reflect less favorable substrate conditions for fungal growth. Previous studies have proposed that increased lignin degradation can be associated with metabolic trade-offs involving carbon and energy allocation toward lignocellulolytic enzyme synthesis [34]; however, this mechanism was not directly evaluated in the present study and should therefore be considered a hypothesis.

At this point, production trends suggest that substrate composition modulates the overall physiology of *P. djamor*, ranging from enzyme synthesis and biomass accumulation to primary and secondary metabolic responses. Consequently, the next logical step is to evaluate whether significant differences occur within its metabolic profile.

### 2.2. Metabolite Profile Analysis

#### 2.2.1. Multivariate Responses to Substrate Gradients

To evaluate global metabolic responses associated with substrate gradients, separate PERMANOVA analyses were performed for coconut fiber and coffee husk formulations using Euclidean distances calculated from the filtered and scaled metabolite matrix.

For coconut fiber-based substrates, inclusion levels explained 8.8% of the total metabolomic variation (R^2^ = 0.088, F = 1.440, *p* = 0.133). Similarly, coffee husk inclusion levels explained 8.0% of the variation in metabolite composition (R^2^ = 0.080, F = 1.216, *p* = 0.225). Although these effects were not statistically significant, these results indicate that substrate gradients accounted for only a modest proportion of the observed metabolomic variability. Homogeneity of multivariate dispersion was evaluated using betadisper analysis. No significant differences in dispersion were detected among coconut fiber treatments (F = 0.132, *p* = 0.982) or coffee husk treatments (F = 1.291, *p* = 0.341), indicating that within-group variability did not influence multivariate comparisons.

Overall, these results indicate that the evaluated substrate gradients did not produce statistically detectable differences in global metabolomic composition under the conditions of this study. The relatively low proportion of variation explained by substrate inclusion (8.8% for coconut fiber and 8.0% for coffee husk), together with the non-significant PERMANOVA results, indicates that the overall metabolomic structure of *P. djamor* remained comparatively stable across the evaluated gradients. Fungi are known to exhibit considerable metabolic plasticity, allowing them to maintain core physiological functions while modulating pathways involved in environmental adaptation and resource utilization [7,34]. Therefore, subsequent PCA, heatmap, and pathway analyses were interpreted as exploratory approaches to examine patterns in individual metabolites and metabolite groups rather than as evidence of global metabolomic differentiation among treatments.

#### 2.2.2. Metabolic Structure and Profiling

To further explore the structure of the metabolomic dataset, principal component analysis (PCA) was performed separately for the coconut fiber and coffee husk gradients. Given the absence of significant global treatment effects in PERMANOVA, PCA was used as an exploratory visualization of sample distribution and metabolite variation rather than as evidence of statistically significant treatment separation (Figure 2).

For coconut fiber-based substrates, the first three principal components explained 60.3% of the total variance, with PC1, PC2, and PC3 accounting for 32.5%, 17.4%, and 10.4% of the variation, respectively. Samples exhibited a gradual distribution across the ordination space along the coconut fiber gradient, with some displacement relative to the barley straw control (Figure 2a). In contrast, coffee husk-based substrates showed a more dispersed distribution across multivariate space. The first three principal components explained 53.2% of the total variance, with PC1, PC2, and PC3 accounting for 23.0%, 21.3%, and 8.9% of the variance, respectively. Samples cultivated on coffee husk formulations occupied a broader region of the ordination space and showed greater dispersion relative to the barley straw control (Figure 2b). In both substrate systems, barley straw samples occupied distinct positions relative to mixed formulations, serving as a metabolic reference for evaluating substrate-associated trajectories.

Consistent with the non-significant PERMANOVA results, PCA did not reveal discrete treatment clusters in either substrate system. Instead, samples showed continuous distributions across the ordination space. Although some gradual patterns were visually apparent along the substrate gradients, these patterns should be considered exploratory and do not constitute evidence of statistically significant global metabolomic differentiation. Subsequent analyses therefore focused on examining the metabolites contributing most strongly to the observed multivariate variation.

To explore which metabolites contributed most strongly to the variation visualized in the PCA, heatmaps were constructed using the 20 metabolites with the highest combined contributions to PC1 and PC2 for each substrate gradient (Figure 3). Distinct metabolite abundance patterns were observed between coconut fiber- and coffee husk-based substrates, revealing coordinated changes in metabolite accumulation along the progressive replacement of barley straw. Although PERMANOVA indicated only modest global metabolomic variation, the heatmaps showed that substrate substitution was associated with consistent changes in specific metabolite groups, revealing the metabolite groups underlying the gradual metabolic trajectories identified by PCA.

The coconut fiber gradient revealed coordinated changes in the relative abundance of several biochemical classes, particularly amino acids, carbohydrates, organic acids, and nitrogen-containing compounds (Figure 3a). Among these, proteinogenic amino acids such as L-methionine, glutamic acid, L-tyrosine, L-valine, and L-asparagine exhibited a progressive decline in relative abundance as barley straw was gradually replaced by coconut fiber. Similar substrate-dependent modulation of amino acid profiles has been reported in *Pleurotus ostreatus* cultivated on different lignocellulosic substrates [35]. Among these metabolites, L-methionine is of particular interest because it serves as the initiating amino acid for protein synthesis and is the precursor of S-adenosylmethionine (SAM), the principal methyl donor involved in numerous biosynthetic and regulatory processes in fungi, including sulfur metabolism, methylation reactions, and cellular growth [36]. Therefore, the variation in the relative abundance of proteinogenic amino acids observed across the coconut fiber gradient may be consistent with localized changes in amino acid metabolism associated with substrate composition. Together with the reduction in biomass yield and biological efficiency observed at high coconut fiber inclusion levels, these patterns may indicate localized metabolic adjustments while maintaining the overall organization of the metabolome. Similar substrate-dependent modulation of amino acid profiles has been reported in *Pleurotus ostreatus* cultivated on different lignocellulosic substrates [35]. L-Methionine is of particular interest because it serves as the initiating amino acid for protein synthesis and is the precursor of S-adenosylmethionine (SAM), a major methyl donor involved in fungal biosynthetic and regulatory processes [36]. However, the present results do not provide sufficient statistical evidence to conclude that substrate substitution altered nitrogen allocation or broadly reprogrammed nitrogen metabolism.

Previous proteomic analyses of *Pleurotus ostreatus* cultivated on different lignocellulosic substrates demonstrated that substrate composition can induce intracellular changes in carbohydrate metabolism and cell wall-associated processes as part of the fungal adaptive response [37]. Consistent with this biological context, N-acetyl-D-glucosamine, a key precursor for chitin biosynthesis and fungal cell wall remodeling, exhibited changes in relative abundance across the coconut fiber gradient. These observations are exploratory, as the present study did not directly assess the underlying regulatory processes.

Coffee husk-based substrates exhibited a distinct metabolite abundance pattern compared with coconut fiber formulations (Figure 3b). The metabolites contributing most strongly to the PCA separation were primarily associated with amino acid metabolism, carbohydrate metabolism, and energy-related intermediates, including alanine, serine, aspartic acid, pyrimidine, ribonic acid, sorbitol, and arabitol. Unlike the coconut fiber gradient, where relative abundances generally declined with increasing substrate substitution, coffee husk formulations displayed more heterogeneous abundance patterns across the substitution gradient, suggesting that progressive incorporation of coffee husk induced differential metabolic responses rather than a uniform increase or decrease in metabolite accumulation.

Coffee husk formulations also exhibited marked changes in the relative abundance of the sugar alcohols arabitol and sorbitol (Figure 3b). These polyols are widely recognized as compatible solutes that contribute to osmotic regulation, oxidative stress tolerance, and environmental adaptation in fungi [38]. Similar responses have been reported in *Endomyces magnusii*, where intracellular arabitol accumulation varied according to glucose availability, indicating that polyol metabolism is closely associated with carbon utilization and environmental conditions [39]. The coordinated variation in polyols observed across the coffee husk gradient may be consistent with changes in carbohydrate-related metabolism. Thus, the observed variation in arabitol and sorbitol provides a potential biological context for the metabolomic patterns associated with coffee husk inclusion; however, the present data do not directly demonstrate altered carbon allocation or enhanced protective carbohydrate metabolism.

Beyond their protective functions, polyols are closely integrated into fungal carbon metabolism. Transcriptomic and genomic studies have demonstrated that carbohydrate utilization is coordinated with amino acid metabolism and acetyl-CoA generation, forming a central metabolic network that regulates carbon allocation toward biomass formation and lipid biosynthesis [40,41,42]. This metabolic connectivity provides a framework for interpreting the lipid-associated metabolites identified in the coffee husk gradient.

Among the lipid-associated metabolites, octadecanoic acid (stearic acid) exhibited marked changes in relative abundance across the coffee husk gradient (Figure 3b). Stearic acid occupies a central position in fungal lipid metabolism, serving as the substrate for Δ9-stearoyl desaturases that generate oleic acid and other unsaturated fatty acids required for membrane biosynthesis and normal fungal development [43]. Experimental disruption of stearoyl desaturase activity in *Aspergillus nidulans* results in stearic acid accumulation accompanied by reduced vegetative growth and sporulation, highlighting the importance of balanced fatty acid metabolism for fungal physiology [43]. Therefore, the variation in stearic acid observed across coffee husk formulations may be consistent with changes in lipid homeostasis associated with substrate composition. The established role of stearic acid in fungal fatty acid metabolism and membrane biosynthesis [43], together with the metabolic connectivity between carbohydrate utilization, amino acid metabolism, and acetyl-CoA generation reported in transcriptomic and genomic studies [40,41,42], provides a biological framework for interpreting these metabolomic patterns. Nevertheless, the present study does not directly establish changes in lipid regulation or carbon allocation.

Together, these findings indicate that substrate substitution was associated with exploratory variation in specific metabolite classes related to amino acid, carbohydrate, and lipid metabolism. To place these metabolite-level changes into a broader biological context, metabolic pathway enrichment analysis was subsequently performed to identify the principal biochemical networks associated with each substrate gradient.

Individual metabolite analysis did not identify statistically significant differences among substrate formulations after multiple-testing correction. Kruskal–Wallis tests were performed separately for the coffee husk and coconut fiber gradients across the 66 metabolites retained for analysis. No metabolite showed an adjusted *p*-value below the significance threshold (FDR < 0.05) in either substrate system. In the coffee husk gradient, Tyrosine showed the lowest nominal *p*-value (*p* = 0.0493), whereas in the coconut fiber gradient, Tyrosine (*p* = 0.0269), 9-octadecenamide (*p* = 0.0370), and L-(-)-arabitol (*p* = 0.0389) showed the lowest nominal *p*-values. However, none of these associations remained significant after Benjamini–Hochberg correction (FDR = 0.7457 for coffee husk and FDR = 0.7015 for coconut fiber). Fold changes relative to the barley straw control were calculated as descriptive measures and were not interpreted as evidence of statistically significant differential metabolite abundance.

#### 2.2.3. Metabolic Pathway Mapping

To place the observed metabolite-level responses into a broader biological context, metabolic pathway analysis was performed to identify the biochemical networks associated with substrate substitution. This approach revealed that the metabolites affected by the coconut fiber and coffee husk gradients were distributed among interconnected pathways related to amino acid, carbohydrate, lipid, and energy metabolism (Figure 4).

Pathway enrichment analysis indicated that amino acid metabolism was among the pathways showing the strongest nominal enrichment in both substrate systems (Table 1). Alanine, aspartate and glutamate metabolism showed the highest pathway impact in both coconut fiber (*p* = 0.004, FDR = 0.2876, impact = 0.327) and coffee husk (*p* = 1.74 × 10^−4^, FDR = 0.0129, impact = 0.450). Thus, this pathway showed statistically supported enrichment after multiple-testing correction in the coffee husk gradient, whereas the corresponding enrichment in the coconut fiber gradient was not significant after FDR adjustment. Several additional pathways showed nominal *p*-values below 0.05; however, most did not remain significant after multiple-testing correction (Table 1). These pathways were therefore considered exploratory rather than statistically confirmed enrichment. The use of a nominal *p*-value threshold for exploratory screening increases the possibility of false-positive pathway associations; therefore, nominal enrichment was not interpreted as confirmatory evidence, and biological conclusions were based primarily on FDR-adjusted results.

Several of the pathways showing nominal enrichment across the substrate gradients were related to amino acid and nitrogen-associated metabolism, including alanine, aspartate and glutamate metabolism, arginine biosynthesis, glutathione metabolism, and nitrogen metabolism (Figure 4; Table 1). However, the statistical support differed among pathways and substrate systems after FDR correction. In particular, alanine, aspartate and glutamate metabolism remained significant after FDR adjustment in the coffee husk gradient (FDR = 0.0129), whereas the corresponding pathway in the coconut fiber gradient did not (FDR = 0.2876). Nitrogen metabolism showed *p*-values below 0.10 in both systems but relatively high FDR values (0.5225 for coffee husk and 0.7482 for coconut fiber) and was therefore considered exploratory. These results suggest potential involvement of amino acid and nitrogen-related metabolism, but they do not provide sufficient evidence to conclude that substrate substitution broadly reorganized fungal nitrogen metabolism.

Nitrogen assimilation is a central process in fungal physiology because glutamate and glutamine constitute the primary nitrogen donors for the biosynthesis of most amino acids through transamination reactions. Consequently, modifications in glutamate-related pathways affect the synthesis and redistribution of numerous downstream amino acids, integrating nitrogen acquisition with protein biosynthesis and cellular metabolism [44]. The observed enrichment of alanine, aspartate and glutamate metabolism is consistent with the variation observed for glutamic acid, alanine, methionine, tyrosine, and asparagine in the heatmap analysis (Figure 3). However, because several of these pathway-level associations did not remain significant after FDR correction, the observed metabolite patterns should be interpreted as exploratory evidence of changes in amino acid metabolism rather than as evidence of altered nitrogen allocation.

Saprotrophic fungi obtain carbon by secreting extracellular enzymes that degrade complex plant polysaccharides, releasing soluble sugars that are subsequently incorporated into central carbon metabolism through glycolysis, the pentose phosphate pathway, the tricarboxylic acid cycle, and the glyoxylate cycle [45]. In filamentous fungi, the expression of polysaccharide-degrading enzymes is tightly regulated by substrate composition and by the carbohydrates released during plant biomass degradation, allowing the fungus to optimize carbon acquisition according to the available lignocellulosic resources [46].

Several carbohydrate-related pathways also showed nominal enrichment across the substrate gradients. Starch and sucrose metabolism presented *p*-values of 0.0215 and 0.0305 for coconut fiber and coffee husk, respectively; however, the corresponding FDR values were 0.5298 and 0.3439 (Table 1). Therefore, these pathway associations did not remain statistically significant after multiple-testing correction and are considered exploratory. The observed variation in trehalose, N-acetyl-D-glucosamine, arabitol, and sorbitol in the heatmap may be consistent with changes in carbohydrate-related metabolism, although the present data do not establish altered carbon allocation or regulation of carbohydrate utilization.

Although several pathways were unique to each substrate, these differences mainly reflected substrate-specific metabolic adjustments superimposed on the common physiological framework described above. Some pathways were observed only in one substrate system, but these differences should be interpreted cautiously because most pathway associations did not remain significant after FDR correction. For example, amino sugar and nucleotide sugar metabolism was observed in the coconut fiber gradient (*p* = 0.0561, FDR = 0.5694), whereas fructose and mannose metabolism and glyoxylate and dicarboxylate metabolism were observed in the coffee husk gradient (*p* = 0.0162 and 0.0525; FDR = 0.3439 and 0.4237, respectively). These results indicate possible substrate-specific metabolic patterns, but they should be considered exploratory rather than statistically confirmed pathway differences.

Overall, pathway analysis provided exploratory associations between the metabolites detected in *P. djamor* and pathways related to amino acid, carbohydrate, and central metabolism. The results did not provide evidence for widespread pathway-level reprogramming across the substrate gradients, particularly after multiple-testing correction. Instead, the pathway analysis identified a limited number of statistically supported associations in the coffee husk gradient together with several additional nominal associations that require further validation. These findings should therefore be interpreted as hypotheses concerning potential metabolic responses to substrate composition rather than as evidence of coordinated metabolic regulation. Together, these results indicate that substrate formulation was associated with differences in mushroom productivity and with exploratory variation in selected metabolite groups and pathway associations. Whether these metabolic patterns contribute directly to differences in fungal physiological performance remains to be determined.

The present study provides a comprehensive overview of the metabolic responses of *P. djamor* to progressive lignocellulosic substrate substitution. However, the metabolomic profiles presented here represent a physiological snapshot and do not directly reveal the regulatory mechanisms controlling the observed metabolic shifts. Future studies integrating metabolomics with transcriptomic, proteomic, and enzyme activity analyses, together with temporal sampling during mushroom development, will help elucidate the molecular regulation underlying nitrogen and carbon metabolism. Such integrative approaches may facilitate the rational design of substrate formulations that simultaneously maximize mushroom productivity, nutritional quality, and the sustainable valorization of agro-industrial residues.

## 3. Materials and Methods

### 3.1. Substrate Preparation

Two agro-industrial residues were used as substrates: coffee husk obtained from local coffee producers and coconut fiber obtained from previous cultivation cycles from greenhouses. All materials were subjected to sun-drying under direct exposure until constant weight was achieved. A total of eleven treatments were prepared using different proportions of agro-industrial residues coconut fiber and coffee husk combined with barley straw as a complementary substrate. The proportions of each residue ranged from 20%, 40%, 60%, 80%, and 100%, with barley added to complete 100% of the substrate mixture. In addition, a control treatment consisting of 100% barley was included. Each treatment was prepared in polypropylene bags (50 × 70 cm) containing 1000 g of dry substrate. Substrates were hydrated for 24 h to reach a final moisture content of 70–80%. Subsequently, the bags were perforated and pasteurized in a 70 L steam system for 2 h. After pasteurization, substrates were allowed to cool at room temperature before inoculation.

The chemical composition of the substrate materials used in this study was not directly characterized in terms of cellulose, hemicellulose, lignin, or carbon-to-nitrogen ratio. Published compositional values are therefore provided only as contextual information for interpretation and do not represent measurements of the specific substrate batches used in the present experiment.

### 3.2. Production of P. djamor

All procedures were conducted under aseptic conditions. Each substrate bag was inoculated at 10% (*w*/*w*) of the moist substrate and supplemented with 5 g of calcium carbonate, which was manually distributed. The colonization phase was carried out in a controlled chamber at temperatures ranging from 13 to 25 °C and relative humidity between 40% and 80%. Once full colonization was achieved, bags were transferred to a fruiting room maintained at 24–26 °C and 70–80% relative humidity under dark conditions [47]. Fruiting bodies were harvested manually when they reached a size greater than 5 cm, using sterile gloves and protective equipment. After harvesting, fresh weight was recorded, and the colonization time was monitored for each treatment. Biological efficiency (BE) (Equation (1)) and yield (Equation (2)) were calculated according to standard methodologies [48] as follows:

(1) $$\mathrm{BE}\;\left(\%\right)=\frac{\mathrm{fresh}\;\mathrm{weight}\;\mathrm{of}\;\mathrm{fruit}\;\mathrm{bodies}}{\mathrm{dry}\;\mathrm{weight}\;\mathrm{of}\;\mathrm{substrate}}\;\times \;100$$

(2) $$\mathrm{Yield}\;\left(\%\right)=\frac{\mathrm{fresh}\;\mathrm{weight}\;\mathrm{of}\;\mathrm{fruit}\;\mathrm{bodies}}{\mathrm{initial}\;\mathrm{wet}\;\mathrm{substrate}\;\mathrm{weight}}\times 100$$

### 3.3. Metabolite Annotation

Metabolite profiling was performed by gas chromatography–mass spectrometry (GC–MS) following the analytical procedure previously established by Cruz-Moreno et al. [47] for *Pleurotus djamor*. Briefly, samples were prepared in HPLC-grade methanol (1 mg/mL), and metabolites were derivatized using BSTFA + 1% TMCS prior to GC–MS analysis. The analyses were performed using an Agilent 7890A gas chromatograph coupled to a 5975C single-quadrupole mass spectrometer equipped with an electron impact ionization source. Helium was used as the carrier gas at 1 mL min^−1^, samples were injected in splitless mode onto an HP-5MS capillary column (30 m × 0.25 mm × 0.25 μm), and mass spectra were acquired over the m/z 50–700 range, following the previously reported analytical conditions [47]. Spectral processing and metabolite annotation were performed using ChemStation (Agilent Technologies, Waldbronn, Germany) software version C.01.10. Acquired electron-impact mass spectra were compared with the NIST 11 mass spectral reference database, and spectral matches with a similarity score greater than 80% were considered for metabolite annotation, following the procedure reported by Cruz-Moreno et al. [47]. Because metabolite assignments were based on mass spectral library matching and were not systematically confirmed using authentic reference standards or retention-index matching, the reported compounds are considered putatively annotated metabolites rather than experimentally confirmed metabolite identifications.

### 3.4. Statistics Analysis

Production variables (yield and biological efficiency, BE) were analyzed at α = 0.05 using three biological replicates per treatment. Data normality and homogeneity of variances were assessed using the Shapiro–Wilk and Bartlett tests, respectively. Depending on data distribution, differences among treatments were evaluated using one-way ANOVA followed by Tukey’s HSD test or the Kruskal–Wallis test followed by Dunn’s test with Benjamini–Hochberg correction.

For metabolomic analysis, three biological replicates were established for each of the 11 substrate formulations, resulting in 33 experimental units. The final metabolomic dataset used for statistical analysis comprised 30 biological samples. Each biological replicate corresponded to an independent cultivation unit. GC–MS metabolomic data were initially filtered using a spectral quality threshold of 70 (Qual ≥ 70), and contaminants were removed based on predefined compound annotations. Peak areas were aggregated by sample and metabolite, and repeated annotations of the same metabolite within a sample were summed. A sample × metabolite matrix was then generated. Metabolites detected in fewer than five samples were excluded using a prevalence filter. Metabolites with variance values at or below the first quartile of the variance distribution were subsequently removed. The remaining metabolite abundances were standardized using Z-score scaling, resulting in a final dataset of 66 metabolites across 30 biological samples.

Global differences in metabolomic composition between substrate formulations were evaluated separately for the coconut fiber and coffee husk gradients using permutational multivariate analysis of variance (PERMANOVA) based on Euclidean distances, with 999 permutations. Homogeneity of multivariate dispersion was assessed using the betadisper procedure. Principal component analysis (PCA) was performed to explore the multivariate structure and distribution of the metabolomic profiles across substrate gradients. Heatmaps were generated using the 20 metabolites with the highest combined contributions to PC1 and PC2 for each substrate system, with metabolite abundances standardized using Z-score normalization.

To complement the multivariate analyses, individual metabolite differences were assessed separately for the coffee husk and coconut fiber gradients using Kruskal–Wallis tests across the 66 metabolites retained for analysis. The tests included the 100% barley straw control and the 20%, 40%, 60%, 80%, and 100% inclusion levels of each alternative substrate. *p* values were adjusted for multiple testing across the 66 metabolites within each substrate using the Benjamini–Hochberg procedure. Fold changes relative to the barley straw control were calculated from median metabolite abundances at each inclusion level and were treated as descriptive measures. An FDR < 0.05 was considered statistically significant.

Finally metabolic pathway enrichment analysis was performed using the Pathway Analysis module of MetaboAnalyst 6.0 (https://www.metaboanalyst.ca/home.xhtml) [49]. Metabolites were mapped against the KEGG database, and pathway enrichment was evaluated by combining over-representation analysis with pathway topology analysis. Nominal *p*-values and false discovery rate (FDR)-adjusted *p*-values were considered in pathway interpretation. A nominal *p*-value threshold of 0.10 was used as a permissive criterion to retain pathways for exploratory evaluation and hypothesis generation, rather than as a threshold for statistical significance. Given the multiple comparisons inherent to pathway enrichment analysis, FDR-adjusted *p*-values were used to control the expected proportion of false discoveries. Pathways with FDR < 0.05 were considered statistically significant, whereas pathways with nominal *p* < 0.10 and FDR ≥ 0.05 were treated as exploratory associations rather than statistically significant enrichment.

## 4. Conclusions

This study demonstrates that progressive changes in lignocellulosic substrate composition had a clear effect on *P. djamor* productivity, while the overall metabolomic organization remained comparatively stable. The metabolomic analyses identified exploratory variation in selected amino acid, carbohydrate, and lipid-associated metabolites, together with limited pathway-level associations, but these patterns did not constitute evidence of widespread metabolic reprogramming. Thus, the main contribution of the study is the demonstration that substantial differences in mushroom productivity can occur without corresponding statistically significant changes in the global metabolomic profile. Further targeted metabolomic, enzymatic, and molecular analyses will be required to determine the mechanisms underlying these responses.

## Figures and Tables

**Figure 1:**
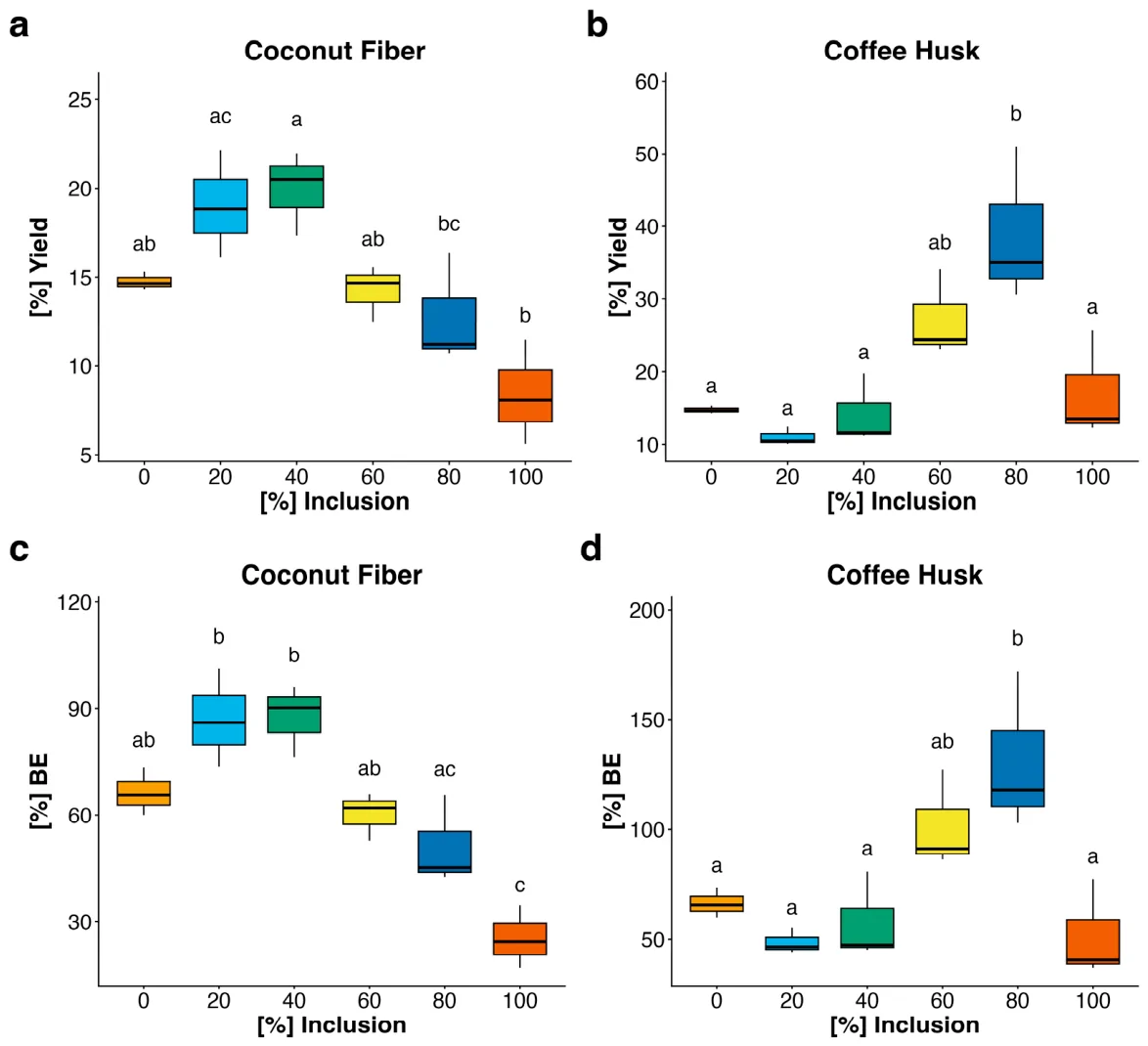
Effect of substrate composition on yield and biological efficiency. (**a**,**b**) Yield (%) and (**c**,**d**) biological efficiency (%) of *P. djamor* cultivated on coconut fiber and coffee husk substrates at different inclusion levels. Different letters indicate significant differences among treatments (*p* < 0.05).

**Figure 2:**
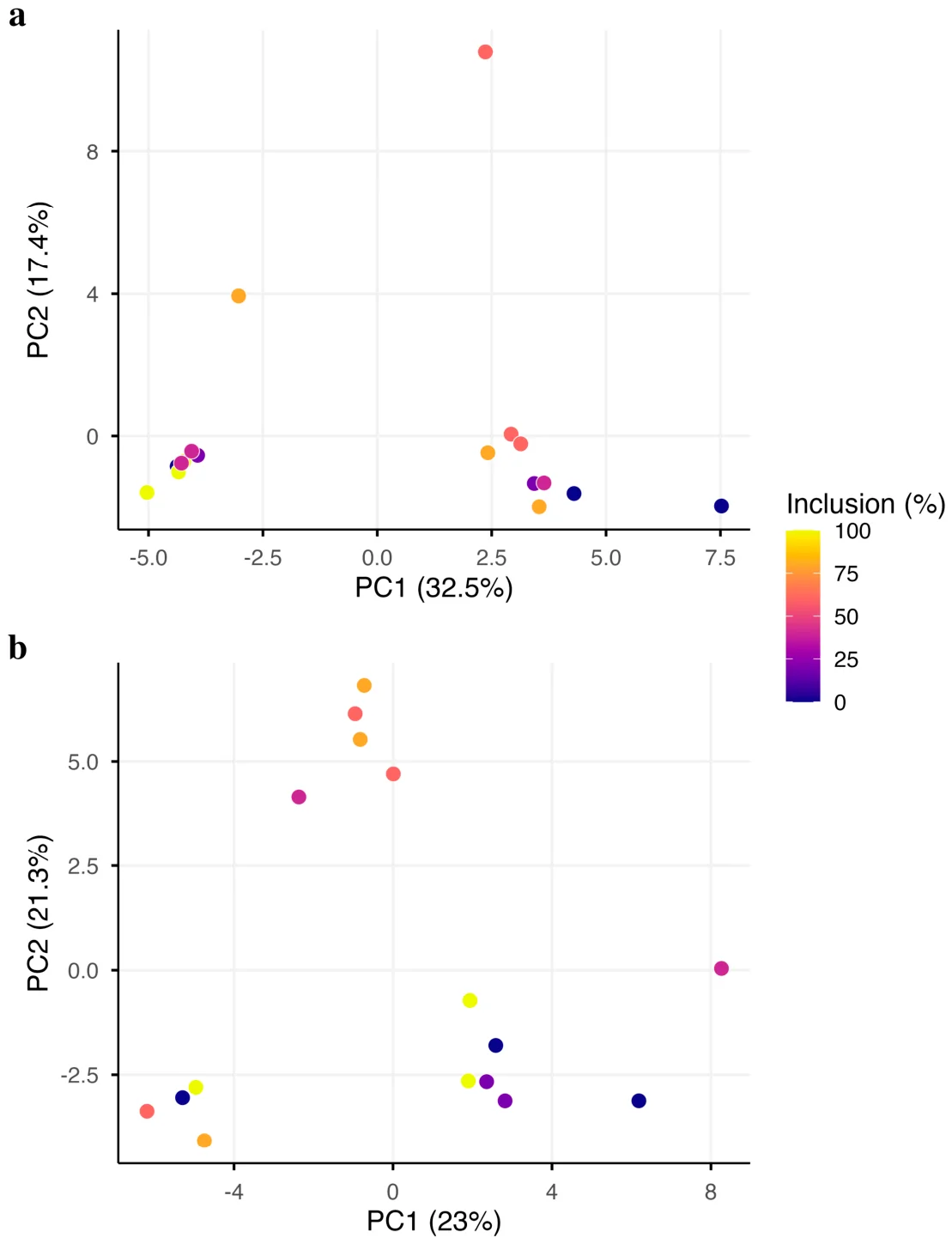
Principal component analysis (PCA) of the GC–MS metabolomic profiles of *Pleurotus djamor* cultivated under progressive substrate replacement gradients. (**a**) Coconut fiber gradient and (**b**) coffee husk gradient. Inclusion levels (20, 40, 60, 80, and 100%) represent the proportion of barley straw replaced by the respective residue, while the barley straw control corresponds to 0% replacement. The distribution of samples is shown to facilitate visualization of exploratory metabolomic variation across the replacement gradients; no discrete treatment separation was observed.

**Figure 3:**
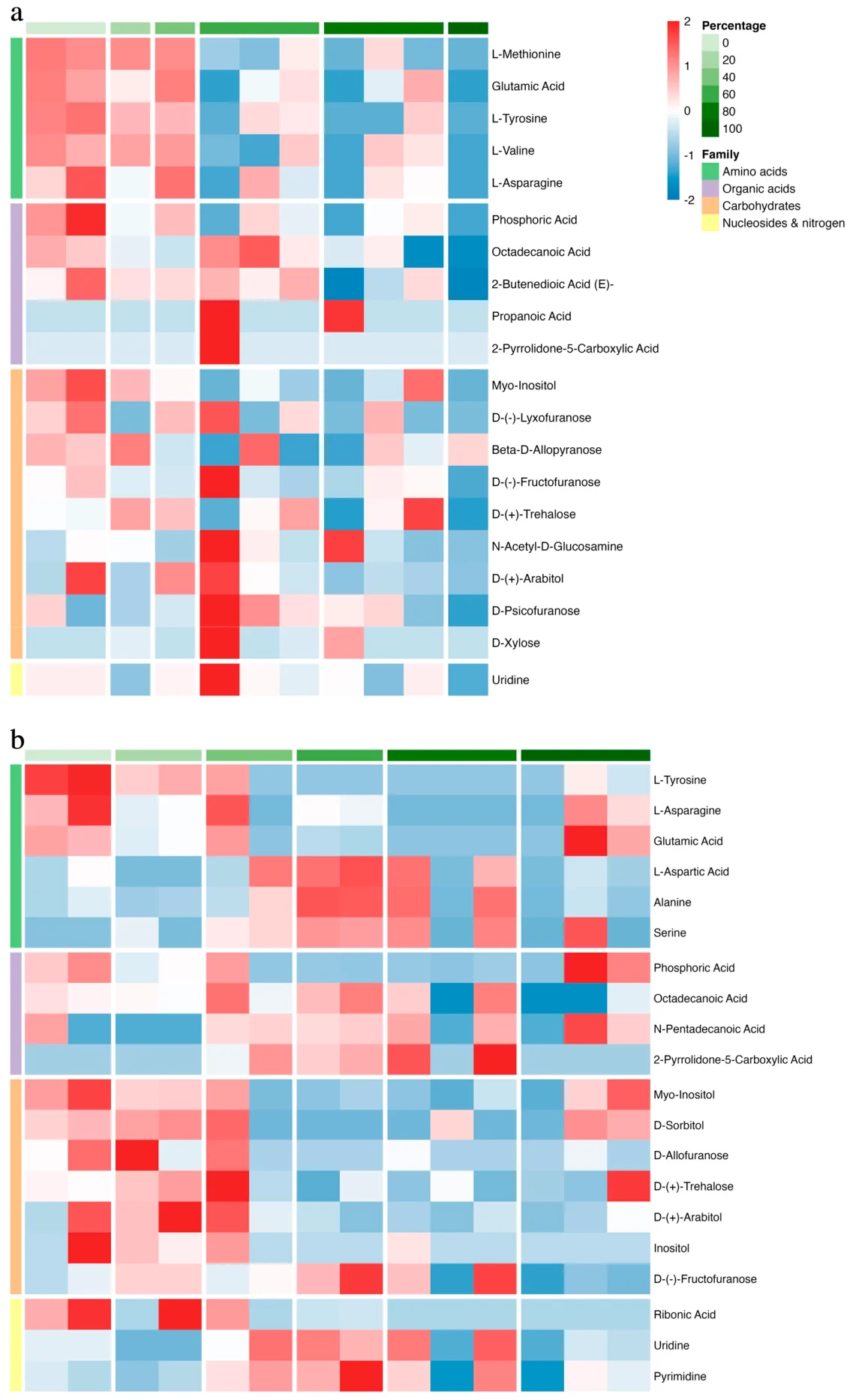
Heatmap of putatively annotated metabolites across the substrate replacement gradients. (**a**) Coconut fiber gradient and (**b**) coffee husk gradient. Columns represent the barley straw control and progressive replacement levels (20, 40, 60, 80, and 100%) with the corresponding residue. The heatmap color scale represents standardized metabolite abundance (Z-score), whereas the metabolite categories indicate compound classes. The figure is intended to illustrate exploratory patterns of metabolite abundance rather than statistically significant differential abundance.

**Figure 4:**
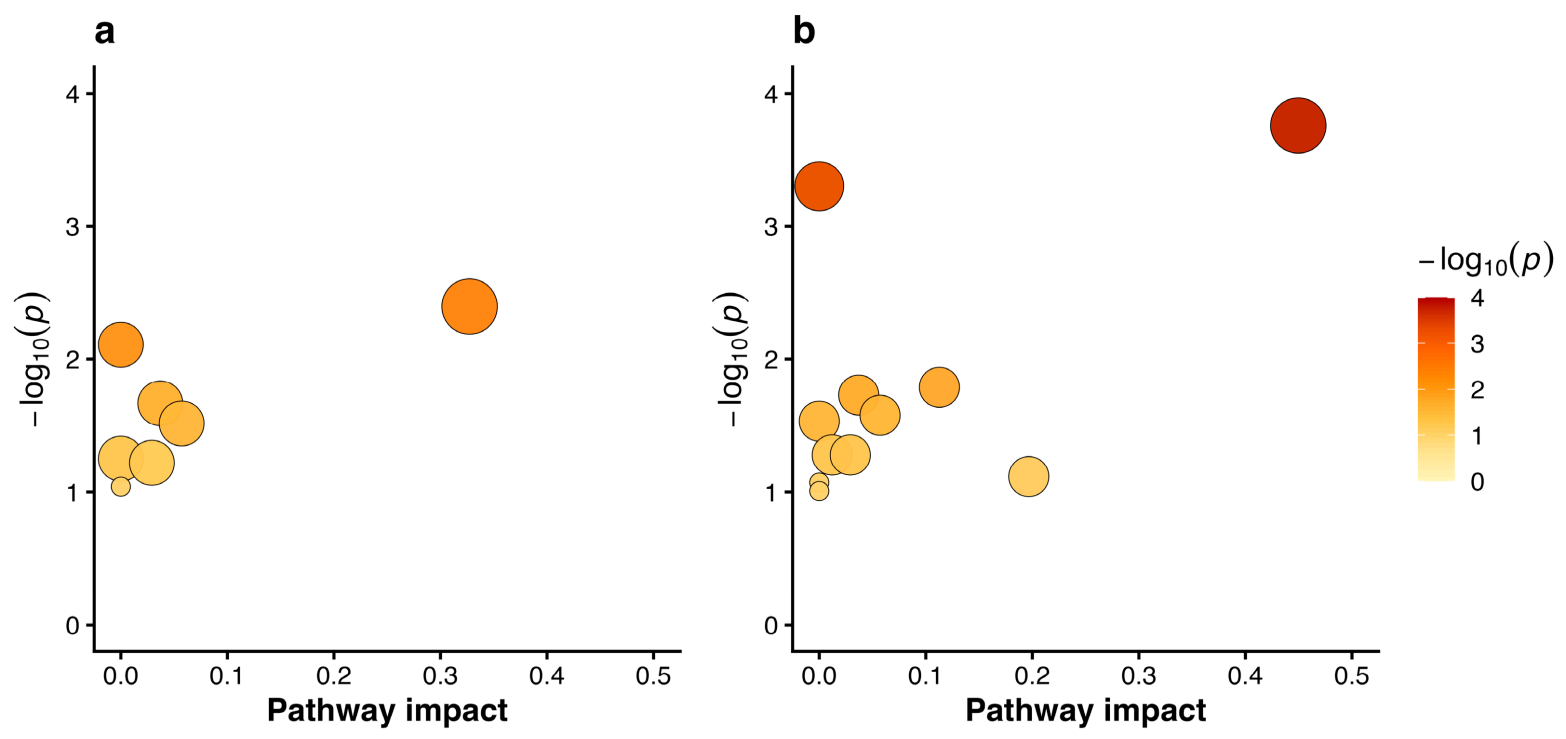
Metabolic pathway analysis of *Pleurotus djamor* across the substrate replacement gradients. (**a**) Coconut fiber gradient and (**b**) coffee husk gradient. The plotted pathways represent associations identified through nominal enrichment analysis. The *x*-axis represents pathway impact, whereas the *y*-axis represents nominal enrichment significance expressed as −log10(*p*). FDR-adjusted *p*-values reported in Table 1 were used to distinguish statistically supported pathway enrichment from exploratory associations. Alanine, aspartate and glutamate metabolism and cyanoamino acid metabolism showed FDR < 0.05 in the coffee husk gradient, whereas the remaining pathway associations were interpreted as exploratory.

**Table 1:** Summary of metabolic pathways showing nominal enrichment in *P. djamor* across coconut fiber and coffee husk replacement gradients, with FDR-adjusted statistical support.

Pathway Name	Coconut Fiber				Coffee Husk			
	Match	*p*	FDR	Impact	Match	*p*	FDR	Impact
Alanine, aspartate and glutamate metabolism	3/22	0.0040	0.2876	0.3273	4/22	1.74 × 10^−4^	0.0129	0.4496
Tyrosine metabolism	2/9	0.0078	0.2876	0.0000	–	–	–	–
Fructose and mannose metabolism	–	–	–	–	2/14	0.0162	0.3439	0.1128
Starch and sucrose metabolism	2/15	0.0215	0.5298	0.0370	2/15	0.0186	0.3439	0.0370
Arginine biosynthesis	2/18	0.0305	0.5635	0.0571	2/18	0.0264	0.3612	0.0571
Cyanoamino acid metabolism	–	–	–	–	3/12	4.98 × 10^−4^	0.0184	0.0000
Amino sugar and nucleotide sugar metabolism	2/25	0.0561	0.5694	0.0000	–	–	–	–
Glyoxylate and dicarboxylate metabolism	–	–	–	–	2/26	0.0525	0.4237	0.0122
Glutathione metabolism	2/26	0.0602	0.5694	0.0291	2/26	0.0525	0.4237	0.0291
Glycine, serine and threonine metabolism	–	–	–	–	2/32	0.0763	0.5134	0.1967
Nitrogen metabolism	1/6	0.0910	0.7482	0.0000	1/6	0.0847	0.5225	0.0000
Taurine and hypotaurine metabolism	–	–	–	–	1/7	0.0982	0.5589	0.0000

Pathways are listed according to nominal *p*-values. FDR-adjusted *p*-values are provided to account for multiple testing; pathways with FDR < 0.05 were considered statistically significant, whereas pathways with higher FDR values were interpreted as exploratory.

## Data Availability

The datasets and R script supporting the findings of this study are openly available in Zenodo at https://doi.org/10.5281/zenodo.22900953.
